# “Capable of much more”: The effects of vocational training on caregiver expectations for neurodivergent dependents in Thailand

**DOI:** 10.1371/journal.pone.0306141

**Published:** 2024-07-17

**Authors:** Drew B. Mallory

**Affiliations:** Sasin School of Management, Chulalongkorn University, Bangkok, Thailand; Birla Institute of Technology and Science, INDIA

## Abstract

Neurodivergence encompasses a spectrum of natural variations in neurological development, including autism, ADHD, and other expressions of cognitive diversity. Caregiver experiences while supporting their neurodivergent dependents critically influence the quality of life outcomes for neurodivergent dependents across life stages, including during the search for work. However, research on caregiver experiences during this stage remains scarce, especially within lesser studied developing contexts like Thailand. This study explored perspectives of Thai caregivers preparing neurodivergent dependents for employment through a focus group (n = 10) of pre-vocational training caregivers and interviews (n = 13) with post-training caregivers. Analysis revealed significant sociocultural factors introducing stigma that affected acceptance, diagnosis, interventions, and policy support. Both groups reported systemic barriers, doubts about future independence, and hopes to increase advocacy and inclusive attitudes. However, caregivers whose dependents had received the training showed marked shifts in their expectations for their dependents’ success. Tailored vocational preparation helping dependents exhibit strengths may transform societal views on neurodiversity from deficiency to natural diversity and enable more meaningful, sustainable futures. These insights elucidate caregiver challenges and aspirations, which can better inform supportive research and interventions in Thailand and other non-Western locales.

## Introduction

*“There are so many judgments in Thailand to make you believe that the child with special needs cannot do anything […] We have to channel our perception first*. *To believe first*.*” (Caregiver FG8M)*

Neurodivergence encompasses a broad spectrum of neurological variations that exist among human beings, illustrating the diverse nature, or neurodiversity, of the human brain. Neurodivergent (ND) variations include conditions such as autism spectrum disorder (ASD), attention deficit hyperactivity disorder (ADHD), dyslexia, and others. It is estimated that neurodivergence affects between 15% and 20% of the global population [1]. Distinguishing between specific ND conditions can be challenging, as many people have undiagnosed or overlapping conditions, especially in under-resourced contexts. Though increasingly accepted as expressions of human neurological diversity, many ND variations are classified as disabilities, in part due to systemic barriers that can impede quality of life.

ND individuals often face difficulties securing employment in adulthood [2] and can rely heavily on caregivers throughout the transition to work [3]. However, caregivers encounter numerous challenges of their own while managing caregiving and other responsibilities [4]. Across studies and contexts, caregivers face unfamiliar territory as dependents reach adulthood in particular, needing to navigate new service systems with reduced access to resources than was previously available [5–7]. They must juggle multifaceted caregiving responsibilities, including medical, social, and financial care while balancing other obligations [4]. Concurrently, they often experience decreased external support from government, school, and other services as dependents reach adulthood, placing additional burdens on caregivers and intensifying challenges [8–10]. This is especially true in resource- or information-poor contexts or when dependents have particular skill deficits [11]. Mothers, often the primary caregivers, describe social isolation, mental health struggles, and declining physical health resulting from years of intensive caregiving, emphasizing the imperative need for social support [12, 13]. These experiences and other caregiver attitudes, significantly impact success chances for dependents. Negative attitudes stemming from societal stigma or doubts about capabilities can become self-fulfilling prophecies, leading to poorer employment and other outcomes [14, 15]. On the other hand, when caregivers have confidence, successful transitions and employment prospects increase [16, 17]. Researchers have called for greater interest in caregivers’ experiences when assessing ND employment outcomes and the consequences of transition and training services [18, 19].

As ND dependents age, caregivers often express the need for guidance on financial planning, managing behaviors, and exploring employment prospects [15, 20, 21]. Crafting post-secondary planning, including enrolling dependents in vocational programs, becomes complex and daunting with limited guidance and support. This lack of direction and a mismatch between opportunities and abilities can lead to feelings of loss, helplessness, and frustration [15, 20]. Although employment outcomes are highly valued, caregivers tend to harbor doubts about employability stemming from societal attitudes or their own feelings of helplessness, which can negatively impact dependents [14]. Meanwhile, transition services, including vocational programs, can help smooth the journey to employment. However, access to these services remains limited, especially when government- or school-sponsored programs are unavailable [22]. In a review of caregiver interventions for stress and anxiety, Agarwal et al. [5] found no interventions explicitly designed for the challenges these caregivers encounter, indicating a persistent need for more research on interventions tailored to address the specific stressors faced by parents of ND transition-aged people.

Some research indicates that caregivers face similar difficulties during this transition stage, regardless of individual differences [23]. Still, with most studies centered on ASD and coming from wealthy Western nations, the applicability of their findings to understudied developing contexts or cases of dependents with other or compound ND conditions is unclear [5]. Although the ND spectrum is broad, research most often focuses on programs for people with ASD alone [24, 25]. This emphasis and the predominant centering of Western contexts with high resources and legal support, where most research on non-pediatric neurodivergence occurs, means critical gaps remain in documenting the true scope of transition experiences for the varied ND individuals seeking work, and the caregivers who support them [5].

In Thailand, evidence on ND transition support remains scarce [26–28]. Most neurodivergence-focused caregiver research has centered on those whose children have ASD—rarely adults. As in Western nations, Thai caregivers of children experience heightened stress, depression, and stigma than their local counterparts [27, 29–33]. Yet they also confront additional challenges, including inadequate government support [34], insufficient education [29], inadequate caregiving knowledge [27], and compromised support networks—all against a backdrop of culturally-ingrained stigma [26, 32, 33]. Like their Western counterparts, they, too, express deep concerns over their dependents’ futures [26]. Almost nothing is known about caregivers of those with other ND conditions.

Despite recent legal advances broadening disability employment opportunities [34], Thailand continues to face insufficient budget allocations for disability services and support, undermining legislation and programming efforts [35, 36]. Merely 15% of the estimated 370,000 individuals with ASD in Thailand have healthcare access [37]. Vocational training programs are nearly unprecedented [28, 38], and employers enumerate long lists of hesitations to hire ND staff [39]. Societal stigma and pressures to hide and mask neurodivergence are vigorously enforced, which further limits opportunities for ND individuals [27]. Further research is critically needed to examine how Thai caregivers of transition-age ND adults view employment preparedness and experience existing vocational services. Such studies can help ensure that services align with caregiver insights and needs. In addition, this research will help address gaps in the literature relating to transitions in non-Western contexts and across the ND spectrum.

Given the scarcity of research on caregiver experiences in Thailand alongside complex societal attitudes toward disability and discrepant governmental, social, and vocational systems [27, 28, 34, 40], it is imperative to establish a baseline of experiences for caregivers of ND people in Thailand on which to build future research. The present study aims to do so by qualitatively exploring the experiences of two stakeholder groups in Bangkok: caregivers of ND dependents not yet transitioned to work and those who have completed vocational training. This study serves as a snapshot of caregiver positioning regarding experiences and support systems. Including both groups in a comparative study allows for comparison of potential shifts in perspectives, needs, and concerns throughout the transition journey. Examining challenges in preparing dependents for work, experiences with services, and expectations for the future contributes to a comprehensive understanding of caregiver experiences during the ND transition in Thailand. Insights into Thai caregiver needs and concerns before and after vocational training can inform better support and policy, ultimately benefiting dependent and caregiver well-being within the country.

## Materials and methods

### Procedure

This study followed Tsui’s [41] guidelines for conducting non-Western organizational research by adopting a hybrid inductive-deductive framework to balance etic and emic sources of knowledge [42]. A reflexive thematic analysis (RTA) with a critical realist approach [43] was chosen to analyze the data qualitatively. Qualitative research can provide insight into participants’ subjective experiences and is appropriate when exploring novel and nuanced topics, aligning with the study’s goal [44]. RTA involves identifying and interpreting patterns in qualitative data through deep and repeated interaction with the content [45]. As a flexible method, it enables the researcher to actively organize latent themes while acknowledging the researcher’s role in interpretation [46]. The critical realist perspective offers a philosophical complement to this method, assuming an objective reality exists beneath participants’ narratives while recognizing that cultural, linguistic, and other factors shape perceptions of that reality. This joint approach can be helpful in situating researcher interpretations in local meanings, which is especially important when covering subject matter foreign to the researcher’s direct experiences or knowledge [41].

Braune and Clarke [43] caution that, while there are differing views on when to engage with relevant literature during RTA, early deep exposure to the subject area may overly narrow analysis. Delaying immersion can enhance inductive analysis. While the researcher held significant subject matter expertise in several relevant areas, prior knowledge about the subject matter of the study was anchored mainly in Western findings. Thus, a middle ground was chosen that facilitated the active challenging of existing ideas by locally identified knowledge [41]. A targeted literature review was first conducted on related topics, such as parental expectations for people with disabilities (PWD) and ND individuals seeking work, with a focus on Southeast Asian and Thai perspectives. Relevant literature was also reviewed on the experiences of families supporting ND individuals completing vocational training programs. Based on this initial review, a list of preliminary questions was developed to explore themes of global and local importance. To ensure topical breadth, the author consulted with local subject matter experts for suggestions on additional influences that may warrant further exploration. To ensure the full scope of caregiver concerns were addressed, a focus group of caregivers with dependents not yet completing a vocational training program was planned to explore pre-transition concerns. The findings from the focus group were incorporated into an expanded protocol for structured interviews with caregivers of dependents who had already completed a vocational training program. This two-step approach resulted in coverage of themes on barriers and enablers to success at work; experiences, expectations, and apprehensions about training programs; relationships; activities outside of work; past work experiences; and knowledge of neurodivergence.

### Participants

Focus group participants included caregivers of ND individuals not yet enrolled in vocational training but who were actively exploring the future work readiness of their dependents. Recruitment occurred between October 22, 2022 and November 1, 2022 through the purposive sampling of an online network for caregivers of people with neurodiverse conditions like ASD facilitated by a community-based advocacy and training provider in Bangkok [47]. Interested caregivers signed up online and completed a brief online survey to assess qualifications. Clinical diagnoses are often difficult to obtain in Thailand. Thus, group participants were invited to self-report their dependent’s neurodiverse status to the best of their knowledge if formal diagnoses were not available. Previous experience regarding obtaining support services was also obtained. Written informed consent was obtained and managed by the partner organization.

Ten caregivers participated in the focus group. Nine identified as parents of dependents with neurodiverse conditions, with one reported a dependent ND sibling. Multiple dependents had overlapping conditions, not all of which had formal diagnoses. Participants ranged in age from 40 to 54, and included eight females and two males. Five were ethnic and national Thai; four were from Western countries (United States, United Kingdom, or Ireland), and one was from Iran. Half identified as expatriates themselves or through their partner’s status. Their professional activities included homemaking, advocacy, and business. Dependents’ characteristics were diverse, with preferred spoken language, nationality, and ethnic makeup often different from their caregivers, reflecting marriages between locals and foreigners, adoption, and broad international experiences.

Interviewees were caregivers of trainees who had completed vocational training within the last three years, recruited through purposive sampling by tne same community-based provider in Bangkok between November 1, 2022 and December 30^,^ 2022 (Liamputtong, 2020). Written informed consent for interviewees was also obtained and managed by the partner organization. This partner organization provides a 2-year certified program to develop skills for future employment via immersive training courses, on-the-job experience, and transition services. The program attempts to place qualifying graduates with internships and jobs. Beyond the training status of dependents, interviewees met the same criteria for diagnosis self-reports, ages, and caregiving relationships as the focus group. All interviewees opted to speak English. Some interviews occurred remotely due to COVID-19. 13 caregivers participated in 11 one-on-one interviews, with two interviews including two caregivers, per participant request. All participants identified as parents of dependents with ND conditions. As with the focus group, about half of the participants reported dependents with overlapping conditions. Interviewees were older on average than focus group participants, ranging from 50 to 75 years old, reflecting the older ages of dependents and additional work experiences. Eight males and five females participated. Only four interviewees were both ethnic and national Thais, although an additional four reported being married to ethnic and national Thais. The non-Thai participants included five from English-speaking, Anglo-Saxon, Western countries, and one from Japan. Their professions included homemaking, business ownership, teaching, and healthcare management. As with focus group participants, dependents’ characteristics were diverse, with preferred spoken language, nationality, and ethnic makeup sometimes different from their caregivers, reflecting marriages between locals and foreigners, adoption, and wide international experiences. Most dependents were full or partial ethnic Thai, often with mixed nationalities.

Caregivers in both groups represented individuals with various ND conditions, including ASD, Down syndrome, attention deficit hyperactivity disorder (ADHD), and others. Interviews also included caregivers of those with additional complex conditions like Williams syndrome or physical complications such as hearing impairment along with other ND statuses. Each interaction began by reviewing privacy rights and seeking voluntary permission to record for educational purposes. Focus group members later exchanged contact details with each other for support. See Table 1 for focus group demographics and Table 2 for interviewee demographics.

**Table 1:** Focus group participants’ demographic information.

#	Age Range	Participant(s) ID	Gender	Years in Thailand	Caregiver Profession	Caregiver Nationality	Caregiver Ethnicity	Dependent Nationality	Dependent Ethnicity	Dependent Primary Neurodivergence
**1**	50–59	FG1F	Female	28	Student, Homemaker	Ireland	White	Ireland	White	ADHD, Dyspraxia, Dyscalculia
**2**	50–59	FG2F	Female	20+	Retired	UK	White	UK	White	ASD
**3**	40–49	FG3F	Male	25	Counseling psychologist	Iran	Asian	Thailand	Asian	Unknown ND diagnosis
**4**	40–49	FG4F	Female	Whole life	PhD student	Thailand	Asian	Thailand	Asian	Tourette’s
**5**	50–59	FG5F	Female	Whole life	Non-profit	Thailand	Asian	Thailand/Australia	Mixed	Down syndrome
**6**	50–59	FG6M	Male	15	Business	USA	White	USA	White	ASD
**7**	50–59	FG7F	Female	Whole life	Undisclosed	Thai	Asian	USA	Asian	ASD
**8**	40–49	FG8M	Female	Whole life	Therapist	Thailand	Asian	Thailand	Asian	Alfi’s syndrome
**9**	40–49	FG9F	Female	7	Undisclosed	UK	Asian	UK	Mixed	ASD
**10**	40–49	FG10F	Female	Whole life	Undisclosed	UK/Thailand	Asian	Thailand	Asian	ASD

**Table 2:** Interviewee’s demographic information.

#	Age	Participant(s) ID	Gender(s)	Years in Thailand	Caregiver Profession	Caregiver Nationality	Caregiver Ethnicity	Dependent Nationality	Dependent Ethnicity	Dependent Neurodivergence
**1**	50–59	I1M	Male	2	Businessman	Japan	Asian	Japan	Asian	ASD
**2**	50–59	I2M	Male	30	Teacher	UK	White	UK/Thailand	Mixed	ADHD, ASD
**3**	50–59	I3M	Male	22	Construcion Management	UK	White	UK/Thailand	Mixed	ASD
**4**	50–59	I4F	Female	Whole life	Homemaker	Thailand	Asian	Thailand	Asian	Hearing impairment, Developmental Delays
**5**	70+	I5M	Male	15	Teacher (Retired)	USA	White	Thailand/USA	Asian	ASD
**6**	60–69	I6M	Male	22	Business owner	New Zealand	White	Australia/Thailand	Mixed	Down syndrome
**7**	60–69	I7FM*	Female/Male	22	Business owners	UK/Australia	White	UK/Australia	Asian (adopted)	Developmental Delays
**8**	50–59	I8FM*	Female/Male	Whole life	Homemaker, Filmmaker	Thailand	Asian	Thailand	Asian	ASD
**9**	60–69	I9M	Male	26	Retired	USA	White	Thailand/USA	Asian	ASD
**10**	50–59	I10F	Female	Whole life	Health Management	Thailand	Asian	Thailand	Asian	Learning Disability
**11**	60–69	I11F	Female	Whole life	Homemaker	Thailand	Asian	Thailand	Asian	ASD, Anxiety

*interview involved two caregivers, who offered complementary perspectives.

### Data analysis

The discussions from the focus group and interviews were automatically transcribed and checked for accuracy by two readers. Filler words were removed to improve readability while maintaining meaning. Next, as described previously, the researcher utilized a hybrid inductive-deductive approach to conduct a critical realist RTA [42, 43]. To ground analysis in local realities rather than imposing etic concepts [41], the researcher first inductively coded and themed the focus group data by systematically identifying and collating features relevant to the context [48]. Local experts provided consultation on cultural and linguistic matters, helping the researcher to refine the focus group codes into reliable preliminary themes [49]. The researcher then deductively coded the interview transcripts using the focus group themes as sensitizing concepts and adding new inductive codes. Coding was continually expanded and refined. As needed, the researcher sought consultation with subject matter experts to obtain alternate perspectives and confirm ambiguities [50]. Given the substantial thematic overlap between the focus group and interview data, the content was then combined for joint analysis to examine convergence and divergence of experiences, after which the data was reassessed as a whole. Finally, to enhance trustworthiness, the researcher further collaborated with local experts in reviewing the entire dataset’s refined themes and thematic map [51, 52].

## Results

Several universal caregiver experiences were identified from the data, which persisted over employment phases and shifted only in focus. Discrepant experiences also materialized, reflecting differences in the dependents’ life stages, the influence of vocational training, and additional contextual factors. Caregivers in both groups also shared a variety of hopes for the future. To explicate the connections between pre- and post-training commonalities and distinctions, caregiver experiences were categorized into six themes, falling under three overarching dimensions: convergent experiences across both groups, divergent experiences between groups, and mixed hopes for the future. Illustrative quotes for each theme and subtheme are provided in the sections below, with “FG” denoting focus group participants and “I” indicating interviewees, followed by the gender(s) of the participant(s) caring for a single ND person. Despite the discrete discussion format used below, themes were deeply interconnected and multifaceted.

### Convergent experiences

Specific experiences were consistent across groups, including issues of culture conflicting with lived experiences and the need for greater support.

### Karma questions: Making sense of fate

During the focus group discussions, the importance of the concept of “karma” emerged spontaneously as a significant topic. In Thailand, karma is a spiritual and philosophical concept deeply rooted in Thai Buddhist traditions. Within this belief system, one’s actions in past and present lives determine one’s present and future circumstances. As FG5F explained, many in Thai society see neurodivergence as a form of karmic punishment: “It starts with a belief system […] You can hear words like, ‘Why is this happening to me? What have I done in my past life that the child is having this condition? Or what has the child done in the past life? Why do I have this child?’ [Social attitudes] are all tangled in the belief system.” In this way, neurodivergence becomes seen as a deserved consequence of past misdeeds, with any associated suffering warranted [53]. Participants claimed that this perspective could leave caregivers with “no hope from the very first day” (FG10F), especially given the perceived fixedness of karma-tied conditions [54]. Believed helplessness leads some Thai family members to “not want to know about” or to deny the condition (FG9F). To this, one non-Thai focus group participant observed that the belief can carry another side as well: “There’s a concept of accepting what is… extreme tolerance. It’s great. But I think there’s a lot we miss when we think ‘there’s nothing we can do but let it be’ and not do anything beyond that.” (FG6M). The participants’ observations echo previous findings linking similar Asian cultural beliefs to stigma against and lower social standing for minority and marginalized groups [55–57].

While traditional karma beliefs initially provided caregivers with a somewhat meaningful explanation of their experiences, interviewees and those in the focus group said they developed more nuanced views over time. For instance, one Thai caregiver suggested that: “another way of looking at it with the same belief system [is] that this is an opportunity for you to make merits for your next life” (FG5F). By reframing having a ND dependent as an opportunity to accrue good karma through the good deeds required in caring for them, traditional karma beliefs could be retained while removing some of the associated negativity. I18F shared a complementary view. Although she first believed her daughter’s neurodivergence was a punishment, her view shifted: “I believe in karma […] But God gave [our daughter] to us. God taught through [her]… We became closer to God because of [her…], which is great.” Shifting perspectives on karma allowed caregivers to impose meaning on otherwise arbitrary conditions.

Others, like I4F, underwent a different change by ultimately learning to avoid the issue as much as possible: “Did he do something bad in his last life? I don’t know. But it’s happening in this life. [People] should deal with it in this life. Don’t bring karma into it […].” This ambivalence was common, as some sought to compartmentalize karma beliefs when faced with unchangeable realities. I10F explained that caregivers “are tired… they want to do something to give them a reason to stop… and put peace in their minds.” I10F had also stopped thinking about things that were unchangeable, even though she continued to hold herself partially responsible for her dependent’s condition: “Why do I need to go back to things that I can’t fix? That’s why I said, instead of spending time thinking about it, I should use that time to do something that would make an impact for the future.” Such perspectives illustrate the complex and changing role of cultural beliefs for caregivers in both groups. By retaining but reframing traditional beliefs, caregivers engaged in meaning-making processes that restored a sense of coherence and control. Even so, however, participants (FG5F, FG6M, FG7F, I4F, I7FM, I8FM, I11F) in both groups said that they and others in Thailand were prone to use karma “excessively” to “put down,” or oversimplify and dismiss, complex issues like neurodivergence (I10F).

### Saving face: Nata and the social politics of neurodivergence

Participants had universally experienced the impact of the linked Thai cultural concept of “face” (*nata*), something FG6M called “a critical element of Thai culture.” I10F noted that *nata* has “tremendous impact” on all those affected by neurodivergence. Broadly, *nata* refers to the sense of social self and public image that individuals precariously construct and lose through their interactions. While the English term “face” refers specifically to public image, the Thai concept of *nata* encompasses a broader sense of social self and identity [58]. Although present across Southeast Asia [56, 57], in Thailand *nata* influences unique social behaviors and decision-making in all parts of life and is often linked to perceived karmic states [59]. Thais thus strive to accumulate *nata* (*dai na*) by maintaining a positive public image and avoiding shame or embarrassment for themselves or family. This often involves concealing situations deemed inappropriate or shameful by societal standards [60]. I4F called this reality “very normal” for caregivers of ND people in Thailand.

Concerns around *nata* affected caregivers more directly than dependents. As FG6M observed, “a child who doesn’t behave in a way that gives their caregivers face […] causes resentment, causes bad feelings.” He expressed frustration with the concept, which he felt was “highly tied into the cultural aspect of autism.” While non-Thais were often less affected than locals, some expatriates still felt the need to conform: “We feel pressure to control our son’s behavior in public to avoid negative reactions from others” (I1M). While dependents themselves were less impacted by *nata* concerns directly, as “the kids are in their own world” (FG10F) and often lacked the social skills to engage in *nata*-related behaviors (I9M), Thai caregivers reported concern over feeling the judgment of others. FG10F explained that “they feel another parent looking at you judgmentally.” Although “[Thai] people are polite… they probably won’t ask” (I11F) directly about dependents’ atypical behaviors in public, participants said Thais were still prone to making assumptions. For example, disruptive behaviors were often taken as indicating low intelligence or poor parenting skills (I5M). I18FM admitted: “The first thing you have to accept is the truth: You are losing face. Yes. And you were losing face all of your life because you have a special child.” This left caregivers, and occasionally dependents, highly sensitive to avoiding additional *nata* loss.

The pressure to preserve *nata* also contributed to the denial of diagnoses (I3M, I4F, I11F) or negative social perceptions of the dependent (I5M). I10F regretted bowing to such pressure early on from her traditional family: “I think if we had done better by accepting sooner that he’s different, maybe we would have done better than this.” She felt that trying to save face for her family may have hurt her son later. However, caregivers were careful to explain that some *nata*-saving behaviors stemmed from pragmatism rather than shame. I4F explained that, given the binary societal view of “neurotypical or disabled” with “nothing on the spectrum,” caregivers were forced to choose which “category” offered the best outcome for their dependent at any given moment. Because “parents in Thai culture feel like something is wrong with their child,” they “worry what people will see.” However, rather than only being concerned for themselves, she explained, caregivers “don’t want their *child* to become stigma[tized].”

Other caregivers chose to address *nata* concerns proactively, confronting assumptions directly. As one foreign caregiver shared: “My [Thai] wife will defend our son and say: ‘This is his autism, not a behavior problem. You don’t understand because you don’t have a child with autism’” (I2M). He noted that she had not always acted in this way, becoming more comfortable over time as she accepted her son’s diagnosis. I11F described reversing potential *nata* loss similarly: “If they don’t know, they would say ‘Why does he act this way?’ So you have to say: he has autism.” Such responses also aimed to avoid stigma rather than shame. While these pragmatic responses could help mitigate *nata* loss, participants emphasized that they often still traced back to prejudice around neurodivergence in Thai society. As I4F explained, “All the saving face—stigma—it all comes under one category.”

### Seeking answers, finding few: Gaps in information and diagnosis

Caregivers faced persistent obstacles accessing appropriate information and services across all life stages, beginning when first recognizing potential neurodivergence in their child. The diagnostic process proved “extraordinarily difficult” (I7FM), requiring exhaustive searches (FG1F, FG4F, I3M, I7FM, I4F, FG9F, FG7F), and heavy reliance on word-of-mouth and internet or informal resources when professional help was inadequate (FG7F, FG2F, FG9F, FG8M, I4F, I8FM, I9M). One caregiver described the precariousness of personal research: “I just googled autism one day and a lot of resources came out and I owe them a lot for that resource” (FG7F). Local and foreign participants noted that expatriates seemed to face unique disadvantages in accessing information, with I6M expressing frustration at the lack of information available in English: “[Caregivers] just need to know where the resources are.” Nevertheless, local language resources were also scarce, even online, almost as if “it doesn’t exist in Thailand” (I3M).

Accessing services also posed difficulties for many reasons. As one caregiver described: “I tried so hard to get in[to services]. I can say that the first ten years, I roamed around for doctors—for service, for therapy” (FG8M). The politics of gaining legitimized “disability status” through medical gatekeeping of expert knowledge was an impassable barrier. FG8M shared his experience, mirroring the stories of others: “The doctors told me that my son has no need for any kind of treatment. They said he will remain bedridden for the rest of his life, so there’s no need.” Some were simply informed their concerns were “imaginary” (I8FM). FG7F explained that many Thais welcome the imposition of clinical judgment over individual needs and lived experiences: “Thai caregivers […] rely so much on doctors [in] seeking information on special needs. They wait for the doctor to tell them what to do. However, the doctors […] primarily focus on physical limitations.” Such adoption of a strict medical model of disability has been shown to be a significant impediment for caregivers of other PWD in Thailand, significantly exacerbating caregiver challenges [61, 62].

Although the participants rejected pathologizing neurodivergence, captured by FG10F stating simply: “It’s not a disease,” the problematic medicalization of dependents and framing of differences as disabilities was compounded by other family members’ unwillingness to acknowledge ND conditions at all. FG9F called this a “dangerous” trend, explaining her situation: “Some family members ‘just didn’t want to know about it,’ which can lead to the failure to access crucial early-stage interventions. Participants believed that the stigma against disability in Thailand, rooted in cultural concepts like *nata* and karma, contributed greatly to this reluctance and lack of public discourse.

While caregivers acknowledged service improvements over the past two decades, listing several medical and community resources (FG1F, FG3F, FG7F, FG8M, FG9F), these still left much to be desired. To compensate, they admitted to gathering knowledge on treatment and support from travel and international friends and relatives (FG1F, FG4F, FG7F), which helped bolster confidence about options but also increased feelings of helplessness and frustration. For example, after attending a conference in the United States, FG4F realized “…there’s therapy there that could help him, but I don’t know how to get there. […] There’s nothing here in Thailand. Everything is elsewhere.” The interviewees acknowledged similar problems in the past but pointed to the training program as helping to resolve many of their unmet needs, described more below. I1M summarized: “The program was very unique, and we cannot look for such a program in another place.”

### Policy pitfalls: Government supports and social stigma

Caregivers’ concerns were compounded by restrictive laws and policies regarding disability and employment in Thailand, particularly impacting foreigners, who were often excluded entirely from protections and services. Focus group members saw a broad lack of support for people with special needs in Thailand, which they linked to the same cultural attitudes described above. FG5F highlighted a discrepancy in reported ASD cases as a sign of willful negligence, stating: “In Thailand, we have about […] 500,000 to 700,000 cases of autism—which is not recorded, of course […]. But the recorded figure by the Department of Statistics is 4,000.” Beyond stigma, she attributed this oversight to financial and social privileges, which allow influential individuals to “not have to think about” legal or policy changes. FG7F spoke for the majority of participants, believing that the lack of change was due to a lack of will: “As far as I know, there are a lot of politicians and [celebrity] influencers in Thailand that have kids with special needs but they choose to hide.”

Interviewees focused more narrowly on employment policies. They highlighted a recent Thai law designed to increase accessibility by mandating that companies fill 1% of their workforce with persons with disabilities. However, in reality, caregivers reported that most companies opted to take a fine rather than comply, while PWD tended to avoid the program due to stigma associated with obtaining a disability verification needed to qualify (I2M, I6M, I9M). This was one reason why so few caregivers pursued formal diagnoses. The disability card enabled access to government programs but had unintended consequences for employment. I11F’s doctor warned that the card would make getting a job more difficult by exempting her son from required military service. Without that service, as I4F explained, “Nobody’s going to hire you” for standard roles, meaning only jobs sponsored by the government would be open. Even if her son applied through conventional channels, the lack of papers would reveal his exemption for autism, re-labeling him as disabled regardless of his abilities. Like I4F, I2M had also not obtained the card for his dependent, indicating his Thai partner saw “it as a stigma” against her and their son.

“Not so much the karma […] but just the stigma” (I5M) was seen as the reason that employers still continued to not hire ND individuals. On the one hand, as I9M described, business owners find it “too much of a bother.” I2M speculated that: “Maybe there’s just not enough of an understanding that people with disabilities or learning disabilities can do the job just as well. There’s an expectation that they won’t be able to.” I2 believed that employers also worried about needed accommodations and finding acceptance in the workplace, something also reported by research [62]. Participants also described concerns over “courtesy stigma,” where the stigma of marginalized groups spreads by association [12]. Such stigma transfer may deter hiring of ND staff to preserve organizational reputation, an analogue for individual-level *nata*.

Years of frustration with limited domestic options or hope for change led some caregivers to seek diagnosis and interventions abroad (FG5F, FG4F, FG6M, FG9F, I3M, I4F). FG1F explained: “I sent my child to the UK rather than Thailand because I felt the UK university was much more able to support his needs—celebrating differences rather than stigmatizing them.” While an extreme option, many had or were currently considering moving abroad permanently for care, education, or employment (FG1F, FG2F, FG4F, FG5F, I1M, I6M, I7FM, I9M). These choices felt more urgent for those of mixed or foreign nationality, whose inability to access resources and social support made them doubt their long-term prospects in Thailand. I7FM had decided to remain for now, though they remained hesitant: “We are seeing more awareness and more recognition and more options now. But I still think that the surface has just been scratched. […] Thailand has a long way to go to help people that don’t fit in the normal expectations of what people think what a human being’s capabilities are.”

### The family paradox: Kinship ties and tensions

Extended family played a complex role in how caregivers managed current and future plans. Both expatriate caregivers and those with families overseas felt especially harmed by the lack of accessible local support, with FG1F highlighting challenges for non-locals: “I think as an expat being here […] you don’t have the support group you have with family. It’s hard, the day-to-day stuff. And being honest, [you don’t have] even the shoulder to cry on.” Caregivers like FG7F without large local families faced the most severe problems, but FG8M said that even caregivers with local relatives often found support both insufficient and ironically limiting: “In Thailand, we have big extended families, so sometimes [our kids] spend more time with their family than with friends,” thus missing essential socialization opportunities.

Many caregivers reported that family members loved and supported them and their dependents regardless of location or involvement level (FG4F, FG5F, FG8M, FG10F, I8FM, I9M, I11F). However, relatives also frequently perpetuated ingrained societal stigma against neurodivergence and were often “ashamed” of ND dependents (I11F). I10F felt that family denial and unrealistic expectations reflected socially intractable norms: “When our grandparents realized that our son is different, I think everyone in this family was in denial… It was very tough for me and my husband to address it.” FG4F described family members labeling and excluding ND dependents who displayed atypical behaviors: “There’s no understanding. Our children are still labeled. […] Other children are told not to be with them, not to play with them.”

These patterns meant that caregivers in both groups held concerns about future care of their dependents in addition to their dependents’ abilities for independence. I7FM remarked: “In the short term, I have siblings that we would look to […]. But [long-term]… we don’t have an answer right now.” Interviewees tended to have more explicit plans involving caregivers and solutions beyond family, but caregivers like I11F did not want to call on relatives forever: “How long can you burden someone?” Reflecting earlier stages of planning, focus group members displayed the most serious doubts about the future, with most agreeing with FG10F, who said she was simply “still not sure” what to expect next.

### Square pegs, round holes: Problems fitting into education systems

Most participants expressed frustration with mainstream public and private schools, of which there were “very, very few options” suitable for their dependents (I7FM). FG1F said that “international schools were very resistant to taking on any child with any challenge” (FG1F). Caregivers described experiences where staff “had no kind of training” (I2M) or “did not want to support” their dependent, thereby failing to accommodate unique learning needs and styles. I4F reported that this meant her son “never learned anything.” Bullying from officials and peers also profoundly impacted dependents (FG10, I6M, I11F), with I3M sharing that it ultimately “crushed” his son and I2M reporting his dependent also “couldn’t make it.” Some positive private school experiences existed, but financially secure caregivers with tended to create their own solutions, like I6M: “We found curriculums for dependents with learning difficulties and implemented them with our own shadow teacher.” While often insufficient, schools were among the only non-family support systems available. This led to post-graduation anxieties, with the focus group worrying about where dependents would go: “When they reach a certain age there’s nothing. Support just disappears” (FG2F).

### Divergent experiences

Significant differences between participants whose dependents had accessed the training program versus those who had not were identified. Caregivers whose dependents finished the training reported far more positive experiences and expectations for the future. The training itself was transformative. Focus group participants, without vocational training exposure, struggled to find solutions and lacked social support. In contrast, interviewees’ analogous concerns appeared to have been largely addressed through the training experience. Fundamental divergences regarding dependents’ skills, caregiver attitudes, and future expectations were also apparent. Focus group members worried about dependents’ social abilities and self-sufficiency. However, interviewees described dramatic improvements in these areas after training. Witnessing dependents thrive also shifted interviewees’ perspectives on their capabilities, often seeming to lead to the adoption of a social versus medical model of ND and disability. This consequently unlocked the development of more concrete visions for interviewees’ dependents’ futures.

### Skills unlocked: Discovering hidden potential

A key difference between groups was the perceived impact of skill acquisition on dependents who completed the program versus those who had not. Focus group participants frequently expressed concerns about dependents’ abilities to get along with others (FG1F, FG2F, FG4F, FG5G, FG6M, FG10F). For instance, FG1F stated: “My biggest issue as a parent is his sociability.” They worried about capacities for relationships, interpreting social cues, and avoiding manipulation. They emphasized challenges in initiating and maintaining friendships due to hesitancy in interacting. FG2F mentioned, “He’s not confident, doesn’t know how to interact socially.” FG10F echoed this, sharing, “He hasn’t always put himself out there and invited them over to play […] he has no other school friends.” In some cases, social avoidance seemed to be a preference, such as with FG4F: “He wouldn’t like the big [social] get-togethers and things like that […] He wouldn’t initiate such things.” However, the consensus among the participants was captured by FG6M, who remarked, “He doesn’t have any friends. He doesn’t have any social contacts outside of the family.” Focus group participants also expressed ongoing concerns about dependents’ practical life skills. They anticipated long-term stress in supporting dependents due to skills deficits: “I personally can see myself for the next 10 to 15 years still getting a screenshot of his bank account every day” due to a lack of financial literacy (FG1F). While some hoped dependents could work part-time in supported roles, most lacked clear expectations for independence (FG1F, FG2F, FG3F, FG5F, FG6F, FG10F). FG2F felt helpless: “He sees no future for himself. How will he ever become independent and deal with the outside world?” (FG2F).

In contrast, caregivers whose dependents completed training reported remarkable transformations in social abilities and the development of daily living skills. I1M described the communication progress of their dependent with language barriers: “Until he joined [the program], he didn’t have much interest in others and didn’t try to start communicating with them. But recently […] he has become more positive and willing.” I1M attributed this to the individualized support of the training program. Low stress and individualized environmental adaptations also led interviewees like I7FM to observe their dependent becoming “much more interactive […] more articulate […] able to have some conversations.” Caregivers directly attributed these transformations to the training program’s support in facilitating dependents’ navigation of complex interpersonal relationships and arising conflict (I1M, I2M, I4F). Continuous applied opportunities to test skills also led to enhanced self-efficacy. I3M explained: “[The program] was really very helpful—incredibly helpful—in giving him that supportive, confident environment to show that, actually, he can do many things very well, and he *can* contribute to an organization” (I3M). I10F also observed: “At [the program], there are many different types of learning differences. I think for him, to see that people are different and not only he has trouble trying to learn or gain some skills. I think it gives him a sense of comfort that he can also learn and achieve something.” I6M added: “Prior to joining [the program], she had no close friends. But whatever was happening with [her] and the training […] communication was hugely beneficial for her confidence.” Like I6M, many interviewees echoed a sense of appreciation to the program, even if they were slightly mystified on how precisely such immense changes had been achieved (I3M, I4F, I7FM, I8FM, I9M, I10F).

### A transformative journey: Evolution of caregiver mindsets

As training experiences from the center transferred home, many caregivers reported observing unexpected shifts in their expectations for the future as their dependents’ abilities progressed. Several interviewees (I3M, I4F, I5M) described shock at witnessing new skills appear in their dependents’ daily activities that they had not thought possible before. For example, I3M experienced profound surprise when he discovered his son engaging in “simple” self-care activities, like cooking an egg, which he had previously believed was beyond his son’s capabilities. Other interviewees noted growth in their dependents’ level of responsibility and independence that challenged their preconceived notions. I11F attributed the increased responsibility she observed in her dependent to having an internship, stating: “He’s become much more responsible. That’s what happens when you have a job.” During their internship, I9M realized that he had been underestimating his dependent for years, seeing that “He learns and observes. And he knows a lot more than we give him credit for.” I6M arrived at the same conclusion: “I realized she was capable of much more than I had thought.” To describe the positive changes they observed, interviewees commonly used words like “growth” (I3M, I6M, I7FM, I10F), “blossoming” (I4F, I7FM), and “more independent” (I1M, I4F, I7FM) as they described rediscovering their dependents’ personalities and interests.

Caregivers’ experiences during the training program powerfully demonstrated the transformative impact of shifting their perspectives away from the medical model of disability, where differences are positioned as innate deficiency and disability. Witnessing participants achieve previously unimagined capacities universally led caregivers to lift their expectations and reflect on how their own prior perceptions and assumptions, grown from social prejudices, may have helped to co-create some of their prior problems. For these caregivers, the successes of the program revealed that their dependents’ previous”disabilities” were not inherent limitations but rather socially constructed by barriers that the supportive training environment helped dismantle. They explicitly acknowledged the impact that their limited acceptance, education, or awareness about their dependents’ capabilities had introduced (I3M, I4F, I6M, I8FM, I9M, I11F). For example, I11F reflected on her own parenting style: “Thai caregivers, they do everything […] basically. That’s why […] he should have been more capable” before the program. I8FM admitted that they initially held the belief that “people with autism don’t have ambition,” but their perspective changed after the program: “[Our daughter] *has* ambition. She has a goal. She’s not perfect, but she knows what she wants to achieve.”

These realizations were not one-sided; caregivers noticed corresponding shifts in their dependents’ self-perceptions (I2M, I3M, I4F, I5M, I7FM, I8FM). I2M described how his dependent’s mood suddenly increased: “He was happier when he was coming home more often. He enjoyed his day. He’d come home, singing. He’d be really happy to be telling me about what was going on and stuff like that.” I9M expressed astonishment at his son’s newfound attitude and independence: “I see his confidence, his happiness. And that he’s developed—where he does everything, and he can go somewhere without us. I mean, that was unthinkable before. It was unthinkable.” They attributed these positive changes to the inclusive environment of the program: “The way they treat their trainees is amazing. It’s the compassion and how they push the kids to do what [the kids] want […] And I feel he’s so happy all the time” (I4F).

## Shifting expectations: Redefining independence

Caregivers in both groups expressed doubts about their dependents achieving complete financial independence or traditional employment. However, they differed in the reasons for this perception and reactions to it. Focus group participants voiced pronounced concerns about future societal integration. They stressed limitations in interests, motivations, and social skills. Most, like FG7F, affirmed unconditionally, committing to “do whatever it takes to have him live independently” but—more importantly—to be happy (FG2F, FG3F, FG4F, FG10F). However, without exposure to solutions, they struggled to formulate concrete plans, remaining unsure about realistic expectations (FG10F) or how to inspire goal-directed actions (FG6M).

While interviewees had similar thoughts, they displayed greater acceptance of dependents’ capabilities and more flexible and positive interpretations of possibilities. I4F’s response was typical: “I’m ashamed to say that, but I think you do go into self-denial sometimes. […] I’ve had to learn to accept that my son does have an issue, and that’s very tough for a parent to do” (I4F). Yet armed with newfound perceived abilities, accepting limitations was now an enabler, allowing I4F to start “thinking outside the box.” Others reported “confidence” and fresh, if adjusted, “hope” for the future (I2M). These new ambitions often involved tempered expectations of total independence and a strengthened focus on well-being. For example, I7FM staid they were now searching for “an environment where maybe she could live and be independent *so she can get that self-esteem*; some work *where she can contribute and enjoy*” (emphasis added). I10F also now focused on emphasizing “self-esteem and the purpose in life,” which she believed was “very important for a person to live with pride.”

Completing the vocational training gave many interviewees more explicit vocational expectations and goals, which they had lacked before. While both focus group and interviewees referred to their dependents’ skills, interviewees were more likely to express expectations that those abilities could now precipitate future success. They directly linked the identification and application of these new capabilities to the hands-on vocational training experiences at the center (I1M, I3M, I4F, I5M, I6M, I8FM, I9M).

Both focus group and interviewees referred to their dependents’ skills; however, interview participants more often discussed skills as tangible strengths, expressing expectations that the dependents’ abilities would precipitate future success. Interviewees often directly linked the identification or application of these capabilities to the applied experiences at the center (I1M, I5M, I6M, I8FM, I4F, I3M, I9M). More than half of the interviewees stated expectations of semi- or full independence in the future, which they mostly had not had before (I1M, I2M, I4F, I6M, I7FM, I8FM, I10F). Several interviewees had been so affected by the program that they now believed that their dependents could pursue vocations replicating the specific food service programs used by the center as practical training opportunities. For I6M, the tangible work experiences proved so beneficial they now planned for their dependent to attain total financial self-sufficiency: “We now envision her managing one or two coffee shops […] The coffee shops will serve as her pathway to financial independence.” Similarly, after their daughter excelled in the program’s culinary training, I8FM expressed excitement about supporting her newfound “ambition” of owning a bakery and launching a unique brand. In contrast, the focus group participants’ limited exposure to potential solutions or examples of successfully achieved independence for dependents like theirs left them perpetually unsure and searching for the external factors that would sufficiently inspire their dependents toward the same goal-directed actions and achievements.

### Envisioning inclusion: Calling for change

When asked about their motivations for participating in the research, both focus group members (FG1F, FG3F, FG4F, FG5F, FG7F, FG8M, FG9F, FG10F) and interviewees (I4F, I7FM, I8FM, I10F, I11F) exhibited strong advocacy and activism tendencies. They expressed a united desire to foster greater societal awareness and catalyze positive social change for individuals with neurodiversity. Focus group participants were more likely to have actively engaged in formal social change initiatives and advocacy activities related to their dependents (FG1F, FG3F, FG4F, FG5F, FG10F) compared to interviewees (I4F, I8FM, I7FM, I11F). They were also more likely to appear to be currently experiencing burnout, like FG9F, who admitted, “I’m really sick of fighting the system.”

Not having yet experienced vocational training programs, focus group members spent their energies on addressing the lack of public neurodiversity-related services and open discourse in the country (FG2F, FG4F, FG5F, FG7F, FG10F). The cost of services was also a point of protest. FG7F explained that for most, “you’ll have a little chance to get into it [good services]. These participants collectively called for action from those in public and powerful positions to improve accessibility and affordability of services. As FG7F stated: “If only this kind of person [would] put the effort to make the service much more available and easier to access with affordable price, it should be better for the kids.” FG9F urged people to speak out: “The more and more people use their voice and come out with hope for their kids, it’s going to change.”

Although interviewees acknowledged that their economic privilege had sheltered them from certain systemic hardships, they also believed they had an obligation to advocate for change. In particular, they wanted to see more opportunities for vocational training and employment for ND individuals from less privileged backgrounds. They recognized that the current training program’s high cost and small scale made it unreachable for most families. Thus, while they focused on incrementally changing societal attitudes by leading through example, they knew real change would require governmental support. As I10F pointed out: “In Thailand, I think awareness of different kids—especially those with learning disabilities—is very low.” To work towards a more inclusive society, interviewees called for a sea change within the public mindset wherein neurodivergence becomes unified with perceptions of ability and value, not deficiency. I4F declared, “The word is going to be their acceptance. That’s the main keyword.” Interviewees expressed hope for greater social acceptance of neurodivergence as a natural aspect of human variation, not a negative trait. They stressed recognizing the capabilities and worth of ND individuals for their differences, not despite them. In this spirit, I4F asserted that others should “Never pity somebody who is neurodivergent or that has a different issue or different kind of a disability.” Over time, just as views on climate change advanced as more public figures engaged in dialogue (I10F), the interviewees hoped that increased advocacy would positively shift perceptions.

All caregivers recognized that attitude changes would take time, and that such changes alone would not suffice without accompanying systemic reforms. I11F said, “it has to to be the policy of the company.” Caregivers believed the Thai government needed to fund substantial improvements in neurodiversity education to help organizations and others “know the results of their [employment] actions” (I10F) while expanding policies that enable meaningful employment. Caregivers from both groups urged other sweeping legal, governmental, and corporate policy changes that could enable the meaningful employment of ND individuals in Thailand, regardless of disability labels (FG5F, FG7F, FG9F, FG10F, I2M, I7FM, I8FM, I10F). They also called for increased government-funded education for employers to promote understanding and acceptance, something currently not prioritized in existing disability legislation or fiscal allocations [34]. Without such systemic efforts, the caregivers believed inclusive attitudes alone would not be enough to create opportunities for marginalized ND individuals or caregivers. I11F pointed out the irony of companies complaining that they cannot find qualified workers with disabilities [62] when people like her dependent were skilled and ready if organizations would: “give them a chance because they have [work experience] in a training program.”

## Discussion

### The influence of sociocultural factors on systemic barriers

This study explored the experiences of caregivers preparing ND dependents for employment in Thailand. Through an exploratory focus group with caregivers of pre-employment dependents and interviews with those whose dependents had completed vocational training, sociocultural factors were identified as the most significant influences on caregiver experiences. Specifically, the Thai conceptualizations of karma and *nata* shaped societal perceptions and acceptance of ND people, including among caregivers [27, 32, 33]. Karma was seen as often promoting resignation towards neurodivergence as an immutable fate, providing a meaningful explanation for caregivers during early stages, but introducing limiting stigma. Caregivers in both groups described shifting perspectives on karma’s link to neurodivergence over time, coming to embrace it as an opportunity for personal growth. As has been found in other Asian countries regarding stigmatized groups, this recasting while remaining within the traditional belief structure appeared to transform neurodivergence into less a deficit than merely a difference [26, 55–57]. However, this perspective may retain some stigmatizing connotations by depicting caring for ND dependents as warranting “good merit” accrued from sacrifice (Ilias et al., 2018). Caregiver emphasis on *nata*, meanwhile, led to feelings of shame and denying diagnoses to avoid stigma, as has been previously documented [34, 40]. Like the response to karma beliefs, some caregivers learned to reframe judgment as reflecting others’ stigma rather than any actual shortcomings. However, the complex societal role of *nata* and the corresponding tendency to penalize non-standard behaviors likely makes resisting demands to conform harder [60].

These sociocultural factors also manifested in systemic obstacles to accessing resources and services. Stigma from karma and *nata* concerns contributed to insufficient public discourse, policy focus, and healthcare resources dedicated to ND conditions [27, 32, 63]. Cultural beliefs also contribute to misconceptions among medical professionals, who overemphasize physical limitations rather than developmental needs, delaying or interfering with diagnosis and proper support [40]. *Nata* sensitivities deterred openly addressing neurodiversity in schools, resulting in problems like bullying, a lack of inclusion, and the absence of transition planning [34]. These culturally rooted forces exacerbated challenges in accessing early interventions, inclusive education, transition services, or disability policies [7, 27, 64]. Those from lower socioeconomic backgrounds faced disproportionate barriers in access to services or support, as they lacked resources to move past stigma barriers. The sociocultural factors of karma and nata also directly impacted workplace stigma and hiring discrimination described by caregivers [27]. For example, the cultural emphasis on conformity and social appearances perpetuated assumptions that ND workers could not adequately perform jobs or fit workplace or educational norms [62]. These beliefs trickled down to workplaces, where the capabilities of ND people were devalued [39].

A unique contribution of this study was the inclusion of expatriate caregivers residing in Thailand, which allowed for a comparison of their caregiving experiences relative to local Thais. Expatriate caregivers faced distinct challenges accessing locally available services, especially those lacking Thai language proficiency and cultural knowledge. They exhibited heavier reliance on resources from their home countries and described pronounced difficulties navigating the Thai healthcare and social service systems. At the same time, local Thai participants voiced similar frustrations regarding limitations in available resources, information, and professional expertise in caring for ND dependents. This suggests that, while expatriate families encounter exaggerated disadvantages due to cultural and language barriers, systemic stigma-driven gaps may fundamentally affect all individuals managing neurodivergence in Thailand.

As a whole, in contrast to some regional reports suggesting a drift away from traditional beliefs regarding neurodivergence, cultural beliefs and systemic problems seem densely intertwined in shaping the caregiving experience for this population in Thailand. The influence of culture appears to remain highly relevant in Thailand for understanding social stigma and systemic barriers faced by ND people across developmental stages, encompassing the employment search [26]. Tackling stigma may thus be a prerequisite for reducing systemic obstacles to improve services and support.

### Intersection of family dynamics in caregiving experiences

Both local and expatriate families experienced complex family dynamics that shaped caregiving experiences. While extended family often provided emotional and practical support, relatives also frequently perpetuated sociocultural stigma against neurodiversity [65]. Assumptions of poor parenting causing conditions led some family members to discourage early diagnosis and intervention. Even when acknowledging conditions over time, ongoing doubts and unrealistic expectations persisted. These mixed influences likely stem from the pervasiveness of Thai cultural beliefs like karma and *nata* in bias against neurodivergence within families and social networks [27].

Sociocultural forces intersected with family dynamics and expatriate status to profoundly shape caregiving experiences. Family plays a complex, influential role in caregiving experiences in Thailand. Although extended family often provided emotional and practical support, relatives also frequently reinforced the prevalent sociocultural stigma against neurodiversity. Assumptions that poor parenting skills cause these conditions led some family members to discourage early diagnosis and intervention. Even when acknowledging conditions over time, ongoing doubts and unrealistic expectations persisted among local and mixed families. These mixed influences likely stem from the pervasiveness of cultural beliefs like karma and *nata* in shaping bias against neurodivergence within families and social networks [27].

For expatriate caregiver families without accessible local relatives, social isolation was notably amplified and mostly unaffected by dependents’ participation in training [66]. At the same time, local Thai participants voiced analogous frustrations regarding limited resources, information and professional expertise. This may suggest that, though expatriates may encounter exaggerated disadvantages, systemic gaps fundamentally affect all families managing ND.

### Strategies for progress

This study suggests systemic gaps in accessible transition services and vocational preparation profoundly impact caregiver experiences preparing dependents for independent living and employment [22]. While those caregivers accessing specialized training saw immense value, they believed quality, affordable training support tailored to ND needs remains scarce in Thailand [28]. Without options, caregivers feel helpless navigating the unknown, unsure how to guide dependents toward optimal vocational directions, as elsewhere [18].

The lack of customized services tailored to ND needs heightens concerns about dependents struggling to meet workplace demands and align skills with opportunities [20]. Expanding access to specialized training services is essential to meet needs, unlock potential, and benefit caregivers. Quality, affordable support tailored to neurodiverse learning needs could help alleviate systemic barriers impacting caregiver readiness and experiences in preparing dependents for employment after graduation. The specialized vocational training program accessed by some caregivers in this study provided customized preparation that closely aligned demonstrated strengths to potential vocational paths, transforming dependents’ and caregivers’ lives [18]. Similar programs have already been studied in Thailand for young people, showing efficacy and family-wide effects [28]. Accessible vocational preparation programs could build skills via customized training tailored to neurodiverse learning needs and meaningful work opportunities [67].

Yet systemic obstacles, stemming partially from prevailing sociocultural attitudes in Thailand that dismiss or underestimate the employment potential of people with neurological differences [27], will require coordinated efforts between stakeholders to implement emerging best practices with sensitivity to Thailand’s unique context [26]. Advocacy and awareness campaigns should aim to transform outdated attitudes about neurodiversity that undermine employment opportunities [68]. Meanwhile, government, schools, vocational programs, and employers must proactively collaborate to foster inclusive environments, explore neurodiverse strengths, and avoid limiting differences [25, 28].

While this study was conducted in Thailand, many of the findings reflect known phenomena regarding transition stages of ND people and caregiver experiences. The effects of *nata* and karma, though unique, parallel broader known issues around stigma, policy, and access. Although reducing stigma appears essential for enabling systemic reforms that provide accessible pathways to self-sufficiency [69], that interviewees independently adopted a social model of disability through seeing their dependents transform through work is noteworthy. This transformation obviated the need for attitude focused interventions. This suggests that directly providing opportunities that allow ND individuals to demonstrate their capabilities may be an effective avenue for attitude change among caregivers and other stakeholders, such as policymakers and organizational leaders. This further underscores the need for an increase in vocational preparation and work opportunities tailored to ND individuals, especially in high-stigma and low-resource environments. Proper implementation of such supportive policies will require continued localized research and tailored legal and policy changes to each context’s distinct social and legal landscape [7, 26, 27]. Including perspectives from diverse stakeholders, such as expatriates and non-locals, is also critical for capturing overlooked needs and ensuring initiatives serve all groups. With coordinated efforts embracing neurodiversity, similar positive impacts could manifest in Thailand and comparable settings where stigma persists.

### Limitations and strengths

The study makes a strong initial effort to explore caregiver experiences in an under-researched cultural context. However, several limitations warrant consideration. The researcher’s positionality as a Western academic inherently shaped interpretations, as this lens informs perspectives rooted in Western scholarship [41]. Moreover, being an outsider and non-Thai speaker likely impacted participant candor on cultural matters [33]. As participants chose their preferred language, language barriers were hopefully reduced. Additionally, to enhance trustworthiness, the researcher extensively collaborated with local experts to help mitigate inherent biases through culturally attuned analysis [14].

Recruiting caregivers through only one organization led to a restricted sample and an overrepresentation of families with substantial financial resources, including expatriates. However, this sample likely mirrors the key demographic realistically accessing comparable services in Thailand. Regardless, the participating caregivers’ accounts provide valuable and rare insights into the privileges and persisting gaps even among the relatively advantaged in Bangkok. However, the small sample size and restricted ranges of social status and cultural/ethnic backgrounds undoubtedly excluded critical perspectives needed to understand the broader Thai context.

The study also has several additional strengths. It answers calls for locally grounded approaches embracing cultural relevance, providing a rare in-depth examination of understudied caregiver groups in Thailand [41]. The insights generated address gaps in scarce literature on neurodiversity in this context [70]. The inclusion of expatriate perspectives offers novelty and sheds light on underexplored challenges they may encounter in Southeast Asia [66]. Exploration of Thai sociocultural constructs around disability builds an important understanding of localized stigma absent in current literature and under-discussed in Thailand due to local taboos [27]. The careful hybrid analysis balanced grounded, local themes with existing research, increasing the findings’ trustworthiness. Above all, the study elucidates caregiver experiences in Thailand to inform future research and culturally attuned interventions supporting neurodiversity in Thailand and Southeast Asia.

## Conclusion

The experiences of caregivers of ND individuals, both pre-and post-accessing a vocational training program in Thailand, reveal complex sociocultural factors that shape acceptance and access to support, mirroring and building upon previous findings in Thailand [27, 32, 33]. Stigma stemming from cultural concepts like *nata* and karma contributes to denial and reluctance to seek early diagnosis and services, as it does in other areas within the region for ND people and other minorities [55–57]. The consistency of these findings across pre- and post-vocational training groups suggests these experiences may be more persistent and widespread than previously thought [26, 57]. Systemic barriers to accessing adequate resources, education, and employment opportunities for ND individuals persist across the lifespan—not just when attempting vocational training [7, 64]. While rare and economically out of reach for many Thais, targeted training programs like the one referenced can transform caregiver perspectives by demonstrating dependents’ potential and employability and may be a more effective method of changing individual and social attitudes than other means [28, 67].
